# Changes in Disability, Severe Disability, and Dependence in Spain (1986–2020)

**DOI:** 10.3389/ijph.2025.1608931

**Published:** 2025-09-29

**Authors:** Javier Casillas-Clot, Andreu Nolasco, Pamela Pereyra-Zamora

**Affiliations:** Research Unit for the Analysis of Mortality and Health Statistics, Department of Community Nursing, Preventive Medicine, Public Health, and History of Science, University of Alicante, Alicante, Spain

**Keywords:** disability, life expectancy, dependence, aging, Sullivan method

## Abstract

**Objectives:**

This study examines changes in disability, severe disability, and dependence in Spain between 1986 and 2020, highlighting implications for long-term care planning.

**Methods:**

We analyzed microdata from four nationally representative disability surveys (1986, 1999, 2008, 2020) and applied the Sullivan method to estimate disability-free, severe-disability-free, and autonomous life expectancy at ages 6, 45, 65, and 85 years, stratified by sex.

**Results:**

Disability-free life expectancy increased in both sexes, with slightly greater gains in men. However, years lived with severe disability and dependence also rose, especially among older adults. Women consistently lived longer but spent more years with disability and dependence than men. These patterns suggest a partial compression of morbidity, concentrated in milder forms of disability.

**Conclusion:**

Spain has experienced a relative compression of disability over the last four decades, accompanied by a growing burden of severe disability and dependence in old age. These trends raise challenges for care systems in aging societies, particularly where informal caregiving remains central. Our findings provide evidence to support health and social policy reforms aimed at building resilient and equitable long-term care models.

## Introduction

The remarkable increase in life expectancy observed across most high-income countries has sparked growing concern about whether additional years of life are accompanied by good health or by increasing levels of disability and dependence. To address this issue, public health research has focused on indicators such as disability-free life expectancy (DFLE), which estimate not only how long people live, but also how many of those years are lived without significant functional limitations [1–3]. These metrics are essential for understanding the evolving needs of aging populations and for anticipating future demands on health and long-term care systems. In this context, analyzing long-term trends in disability and dependence at the national level provides valuable insights into the sustainability of care models and the equity of support systems, particularly in societies facing rapid demographic aging.

In public health, disability is typically understood through the International Classification of Functioning, Disability and Health (ICF), which defines it as a result of impairments, activity limitations, and participation restrictions caused by the interaction between health conditions and contextual factors [4]. Dependence, in turn, refers to the need for personal assistance or technical aids to perform basic or instrumental activities of daily living. These concepts are particularly relevant in aging societies, where the distribution and severity of disability have direct implications for long-term care needs [5].

While international indicators such as Healthy Life Years (HLY) are widely used for cross-country comparisons, they are based on a single self-reported item and do not distinguish the severity or functional impact of limitations. [3]. Spain regularly contributes to these international datasets, yet such harmonized metrics may overlook national specificities in the distribution and intensity of disability. As a result, complementary national-level analyses remain essential for understanding population needs, especially in contexts where care systems are under pressure and long-term planning depends on accurate projections of dependency and support requirements.

Since the 1980s, several theoretical models have emerged to explain how gains in longevity might relate to trends in disability. Gruenberg’s “failures of success” hypothesis argued that medical advances had reduced mortality without preventing the onset of chronic or disabling conditions, thereby extending years lived with disability [6]. Fries’ compression of morbidity theory suggested the opposite—that better health behaviors and preventive care could delay the onset of disability, compressing it into the final years of life [7]. Manton’s dynamic equilibrium model offered a middle ground, where disease prevalence may remain stable or rise, but with a shift toward milder forms of disability [8].

Empirical evidence has since revealed heterogeneous patterns. While some countries have reported compression of disability, others have seen expansion, or shifting thresholds between mild, severe, and dependent states [9, 10]. These divergences reflect not only epidemiological differences (e.g., morbidity profiles, health behaviors), but they also have important implications for care provision. In Southern European countries, including Spain, where a familialist model of care predominates and informal caregiving by family members—especially women—remains central [11], rising disability prevalence may put additional pressure on households. This pressure is further compounded by demographic and social changes, such as population aging, declining fertility, rising female labor force participation, and the growth of single-person households [12].

Spain, despite having one of the highest life expectancies globally, has limited long-term evidence on national trends in disability, particularly regarding severity and dependence. Earlier research has documented both increases in chronic conditions [13, 14] and signs of compression in functional limitations [15, 16], but often with methodological or geographic limitations. Moreover, little is known about how the burden of disability has evolved by age, sex, and intensity of limitation over the past three decades.

Understanding these trends is essential for anticipating care demands and evaluating the sustainability of current models of provision. While the majority of dependent older adults in Spain still rely on family members for support, recent evidence points to a slow but growing shift toward defamilialization, particularly among the oldest age groups [17, 18]. Mapping the evolution of disability, severe disability, and dependence is therefore not only an epidemiological task, but also one with major implications for care systems and policy planning.

This study examines trends in total and disability-free life expectancy, as well as in years lived with severe disability, and with dependence, in Spain between 1986 and 2020. Drawing on four waves of nationally representative disability surveys and applying the Sullivan method, we analyze how these indicators have evolved over time by age and sex. Spain represents a particularly relevant case for studying these dynamics, as it combines one of the highest life expectancies in the world with a traditionally family-based model of care provision. While not aiming to compare countries directly, our findings contribute to broader public health discussions on aging, disability, and care, offering insights that may inform policy responses in other aging societies where informal caregiving continues to play a central role.

## Methods

This study used microdata from four cross-sectional disability surveys conducted in Spain in 1986, 1999, 2008, and 2020: the *Encuesta sobre Discapacidades, Deficiencias y Minusvalías* [19], the *Encuesta sobre Discapacidades, Deficiencias y Estado de Salud* [20], and the *Encuesta sobre Discapacidad, Autonomía personal y Situaciones de Dependencia* [21, 22]. All four surveys were designed and carried out by the Spanish National Statistics Institute (INE) to estimate the prevalence and severity of disability in the population aged 6 years and older living in private households. The core samples excluded institutionalized individuals, although supplementary institutional samples were collected in 2008 and 2020. To ensure comparability across the entire 1986–2020 period, these supplementary samples were not included in our analysis. Each survey used probabilistic, stratified sampling methods to ensure national representativeness.

### Disability Definitions and Harmonization

The conceptualization of disability has evolved over the study period, moving from the International Classification of Impairments, Disabilities, and Handicaps (ICIDH, 1980) to the International Classification of Functioning, Disability and Health (ICF, 2001), with partial alignment to WHODAS 2.0 (World Health Organization Disability Assessment Schedule 2.0) in later surveys. To allow comparability over time, we harmonized definitions of disability, severe disability, and dependence across the four surveys. Full harmonization details, including item wording, coding schemes, and justifications for comparability, are provided in the Supplementary Material (Supplementary Text A1, Supplementary Tables S1, S2).

In line with the ICF [4], disability was defined as reporting at least one difficulty in performing basic activities of daily living (e.g., walking, dressing, seeing) due to a long-term health problem (lasting or expected to last ≥12 months). Severe disability was defined as being unable to perform at least one of these activities. Dependence was defined as needing personal assistance or technical aids to carry out those tasks.

Two operational measures of disability were constructed. The first is a harmonized definition, restricted to functional domains available and comparable across all four surveys (vision, hearing, mobility, and self-care). The second is a broader definition, incorporating all available domains, severity gradations, and dependence indicators; this version excludes the 1986 survey due to lack of compatible data. Although WHODAS 2.0 was not fully implemented, later surveys were conceptually aligned with the ICF and were explicitly designed by the survey team to preserve longitudinal comparability with earlier waves. This was achieved through specific modifications to item content and structure—for example, retaining domains such as vision and hearing as functional areas—ensuring that core constructs remained comparable over time. These decisions aimed to balance conceptual robustness with continuity.

### Prevalence and Life Expectancy Estimation

We calculated age- and sex-specific prevalence rates of disability, severe disability, and dependence using the survey weights provided by INE. These prevalence estimates were applied to abridged period life tables from INE to estimate total and health expectancies at ages 6, 45, 65, and 85. These ages were selected to represent meaningful life stages and are commonly used in international studies on disability trends.

### Sullivan Method

Health expectancies were estimated using the Sullivan method [23, 24], which combines age-specific prevalence data with life table mortality to partition total life expectancy into years lived with and without health problems. The same approach was used for disability, severe disability, and dependence. This method provides robust population-level estimates and is widely used in international comparisons. Confidence intervals were calculated following the standard variance formulas published by the European Health Expectancy Monitoring Unit (EHEMU) [25].

In addition to estimating absolute values of life expectancy and health expectancies, we calculated relative growth rates (i.e., percentage change between time points) for each measure by age and sex, in order to capture the magnitude and direction of changes over time. We also estimated the proportion of total life expectancy lived with and without disability, severe disability, and dependence, to assess whether gains in longevity were accompanied by compression or expansion of morbidity. Given the complex sample design of the surveys, all estimates were weighted using the sampling weights provided. Statistical analyses were conducted using SPSS v.25^®^.

## Results

A clear demographic shift is observed in the Spanish population, with the largest age group moving progressively from ages 10–14 in 1986 to 40–44 in 2020. The proportion of the population in older age groups has also increased substantially over time (Supplementary Figure S1). At the same time, the distribution of disability by age and sex shows parallel changes across the study period.

In relative terms, disability rates consistently rise with age in both men and women across all periods. However, changes in population age structure have also resulted in notable shifts in the structure of disability. In 1986, the highest number of people with disabilities was found in the 60–64 age group for men and 70–74 for women. By 1999, this shifted to the 75–79 group for both sexes. In 2008, the peak remained at 75–79 for men but rose to 80–84 for women. Finally, in 2020, the highest number of people with disabilities was observed in the 80–84 age group for men and 85–89 for women.

### Life With and Without Disabilities

Table 1 shows life expectancy (LE), disability-free life expectancy (DFLE), and years lived with disability (DLY) at ages 6, 45, 65, and 85 for the total population, men, and women in 1986, 1999, 2008, and 2020. DFLE at younger ages reflects the potential for healthy years ahead, while at older ages it serves as an indicator of age-related morbidity and the degree of compression or expansion of disability.

**TABLE 1:** Life Expectancy, Disability-free life expectancy and years with Disability with 95% Confidence Intervals at 6, 45, 65 and 85 years old in 1986, 1999, 2008 and 2020 (Spain, 1986–2020).

	Life expectancy				Disability-free life expectancy^a^				Years lived with disability^a^			
Age	1986	1999	2008	2020	1986	1999	2008	2020	1986	1999	2008	2020
Total												
6	71.63	73.33	75.64	76.69	62.92 (62.14–63.65)	65.43 (65.10–65.76)	68.09 (67.77–68.41)	69.41 (69.03–69.78)	8.71 (7.98–9.49)	7.90 (7.57–8.23)	7.55 (7.23–7.87)	7.28 (6.91–7.66)
45	34.19	35.72	37.61	38.36	25.75 (25.32–26.19)	28.36 (28.10–28.62)	30.58 (30.33–30.83)	31.55 (31.27–31.84)	8.44 (7.87–8.87)	7.36 (7.10–7.62)	7.03 (6.78–7.28)	6.81 (6.52–7.09)
65	17.20	18.34	20.02	20.53	10.13 (9.88–10.38)	11.95 (11.76–12.13)	13.91 (13.72–14.10)	14.63 (14.42–14.84)	7.07 (6.82–7.32)	6.39 (6.21–6.58)	6.11 (5.92–6.30)	5.90 (5.69–6.11)
85	5.55	5.67	6.35	6.71	1.37 (1.27–1.47)	2.21 (2.13–2.30)	2.61 (2.52–2.70)	2.71 (2.62–2.80)	4.18 (4.08–4.28)	3.46 (3.37–3.55)	3.74 (3.65–3.83)	4.00 (3.91–4.09)
Male												
6	68.43	69.88	72.58	73.95	61.47 (60.46–62.48)	63.98 (63.52–64.43)	66.89 (66.47–67.32)	68.30 (67.81–68.78)	6.96 (5.95–7.97)	5.90 (5.45–6.35)	5.69 (5.26–6.11)	5.65 (5.17–6.14)
45	31.45	32.74	34.82	35.78	24.74 (24.16–25.33)	27.53 (27.19–27.88)	29.70 (29.38–30.03)	30.62 (30.26–30.98)	6.71 (6.12–7.29)	5.21 (4.86–5.56)	5.12 (4.79–5.45)	5.16 (4.80–5.52)
65	15.32	16.17	17.94	18.45	9.71 (9.35–10.06)	11.77 (11–51–12.03)	13.56 (13.31–13.82)	14.11 (13.84–14.39)	5.61 (5.26–5.97)	4.40 (4.14–4.66)	4.38 (4.12–4.64)	4.34 (4.06–4.61)
85	5.07	5.02	5.66	5.99	1.52 (1.35–1.70)	2.33 (2.17–2.49)	2.79 (2.64–2.94)	2.86 (2.72–2.99)	3.55 (3.37–3.73)	2.69 (2.53–2.85)	2.87 (2.72–3.02)	3.13 (2.98–3.28)
Female												
6	74.74	76.79	78.65	79.42	64.32 (63.20–65.45)	67.68 (67.17–68.20)	69.32 (68.84–69.80)	70.49 (69.94–71.05)	10.42 (9.29–11.54)	9.11 (8.60–9.62)	9.33 (8.85–9.81)	8.93 (8.37–9.48)
45	36.74	38.61	40.29	40.91	26.72 (26.08–27.35)	30.10 (29.70–30.51)	31.45 (31.07–31.84)	32.45 (32.02–32.89)	10.02 (9.39–10.66)	8.51 (8.11–8.91)	8.84 (8.46–9.22)	8.46 (8.02–8.90)
65	18.73	20.23	21.83	22.45	10.48 (10.13–10.83)	12.94 (12.64–13.23)	14.22 (13.94–14.50)	15.08 (14.76–15.40)	8.25 (7.90–8.60)	7.29 (7.00–7.58)	7.61 (7.33–7.89)	7.37 (7.05–7.69)
85	5.80	6.01	6.71	7.16	1.29 (1.17–1.41)	2.35 (2.22–2.47)	2.49 (2.38–2.61)	2.60 (2.48–2.72)	4.51 (4.39–4.63)	3.66 (3.54–3.78)	4.22 (4.10–4.34)	4.56 (4.44–4.68)

^a^ Harmonized disability measure to allow comparability with the 1986 survey.

Overall, both life expectancy (LE) and disability-free life expectancy (DFLE) increased steadily across most ages and survey years. At age 6, DFLE increased from 62.9 years in 1986 to 69.4 years in 2020, while years lived with disability (DLY) decreased from 8.7 to 7.3 years. At age 65, DFLE rose from 10.1 to 14.6 years, and DLY decreased from 7.1 to 5.9 years. Similar trends were observed at other ages, with higher DFLE and lower DLY in later years compared to earlier ones. However, several recent changes—particularly the reductions in DLY at ages 6, 45, and 65 between 2008 and 2020—were small and not statistically significant (overlapping 95% CIs). At age 85, the change in DFLE between 2008 and 2020 was also not significant.

Women consistently lived longer than men and had higher DFLE at all time points. In 2020, women aged 65 had a DFLE of 15.1 years compared to 14.1 years for men. However, women also had more years lived with disability: 7.4 years versus 4.3 years in men. At age 85, DFLE increased for both sexes from 1986 to 2020 (from 1.4 to 2.7 years), while DLY remained stable in women and increased slightly in men.

Table 2 presents absolute and relative changes in LE, DFLE, and DLY between 1986 and 2020, as well as sex differences by age and year. LE increased at all ages, with the largest absolute gain at age 6 (+5.06 years) and the smallest at age 85 (+1.16 years). DFLE increased more sharply than LE, especially at age 85, where it rose by 1.34 years (a 97.8% increase). DLY decreased across all ages, with the largest absolute reduction at age 45 (−1.63 years) and the smallest at age 85 (−0.18 years).

**TABLE 2:** Changes in Life Expectancy, Disability-free life expectancy, and years with Disability (1986–2020) with female-male differences by age and year (Spain, 1986–2020).

	Δ2020–1986^a^			ΔDFLE: Female-Male^b^				ΔDLY: Female-Male^b^			
	LE	DFLE	DLY	1986	1999	2008	2020	1986	1999	2008	2020
6	+5.06 (7.1%)	+6.49 (10.3%)	−1.43 (−16.4%)	+2.85 (4.6%)	+3.70 (5.8%)	+2.43 (3.6%)	+2.19 (3.2%)	+3.46 (49.7%)	+3.21 (54.4%)	+3.64 (63.9%)	+3.28 (58.1%)
45	+4.17 (12.2%)	+5.80 (22.5%)	−1.63 (−19.3%)	+1.98 (8.0%)	+2.57 (9.3%)	+1.75 (5.9%)	+1.83 (6.0%)	+3.31 (49.3%)	+3.30 (63.3%)	+3.72 (72.7%)	+3.30 (64.0%)
65	+3.33 (19.4%)	+4.50 (44.4%)	−1.17 (−16.5%)	+0.77 (7.9%)	+1.17 (9.9%)	+0.66 (4.9%)	+0.97 (6.9%)	+2.64 (47.0%)	+2.89 (65.7%)	+3.23 (73.7%)	+3.03 (69.8%)
85	+1.16 (20.9%)	+1.34 (97.8%)	−0.18 (−4.3%)	−0.23 (−15.1%)	+0.02 (0.9%)	−0.12 (−4.3%)	−0.26 (−9.1%)	+0.96 (27.0%)	+0.97 (36.1%)	+1.35 (47.0%)	+1.43 (45.7%)

^a^ Δ Growth rate presented in absolute terms with the relative growth rate shown in parentheses.

^b^ Male-Female differences use males as the reference group for each age and year; LE, Life Expectancy; DLFE, Disability-Free Life Expectancy; DLY, Years with disability.

Across the study period, females consistently had higher DFLE than males. The female–male gap in DFLE narrowed slightly at younger ages (e.g., from +2.85 years at age 6 in 1986 to +2.19 years in 2020), while remaining stable or slightly widening at older ages. The female–male gap in DLY persisted throughout the period. In 2020, women aged 6 lived 3.28 more years with disability than men of the same age.

**FIGURE 1:**
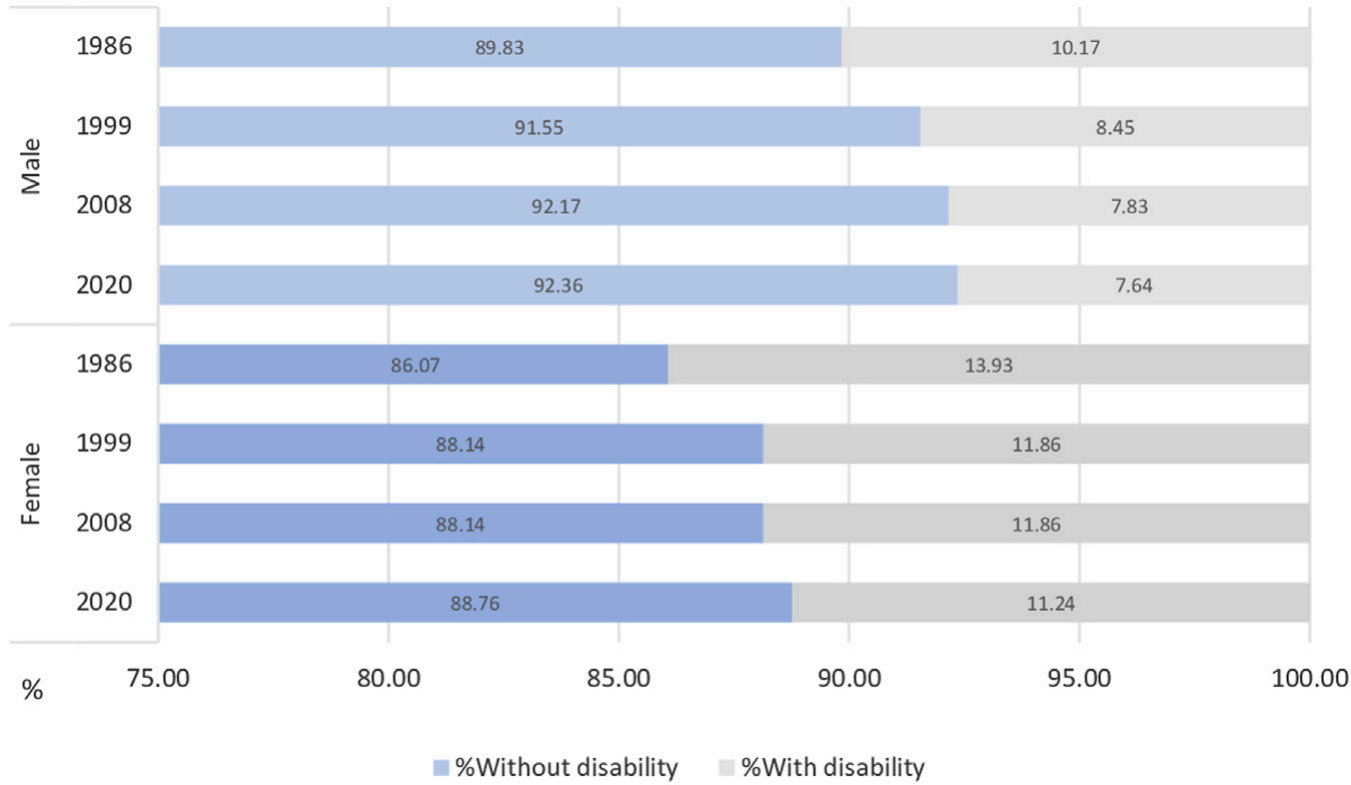
Percentage of life expectancy at 6 years of age lived without disability, by sex in Spain in 1986, 1999, 2008 and 2020 (Spain, 1986–2020).

Figure 1 displays the proportion of total life expectancy spent with and without disability at age 6 by sex and year. Over time, the proportion of DFLE increased in both sexes. Men consistently had a higher share of DFLE relative to their total life expectancy than women.

### Severe Disability and Dependency

Table 3 presents disability-free life expectancy (DFLE), severe disability-free life expectancy (SDFLE), and autonomous life expectancy (ALE) at ages 6, 45, 65, and 85 for 1999, 2008, and 2020, with 95% confidence intervals and estimates of absolute and relative changes.

**TABLE 3:** Life expectancy without Disability, Severe Disability and with Autonomy with 95% Coefficient Intervals at 6, 45, 65 and 85 years old in 1999, 2008 and 2020 (Spain, 1999–2020).

	Disability-free life expectancy				Severe disability-free life expectancy				Autonomous life expectancy			
	1999	2008	2020	Δ1999-2020 (abs; %)^a^	1999	2008	2020	Δ1999-2020 (abs; %)^a^	1999	2008	2020	Δ1999-2020 (abs; %)^a^
Total												
6	64.90 (64.53–65.27)	67.23 (66.88–67.57)	68.18 (67.77–68.59)	3.28 (5.06%)	68.82 (68.54–69.11)	71.41 (71.16–71.67)	71.72 (71.41–72.03)	2.90 (4.21%)	67.96 (67.65–68.26)	69.81 (69.52–70.10)	70.06 (69.70–70.42)	2.10 (3.09%)
45	28.13 (27.84–28.41)	29.99 (29.72–30.25)	30.68 (30.39–30.98)	2.55 (9.07%)	31.59 (31.37–31.82)	33.71 (33.51–33.91)	33.75 (33.52 (33.98)	2.16 (6.84%)	30.82 (30.59–31.06)	32.21 (31.98–32.44)	32.30 (32.03–32.56)	1.48 (4.80%)
65	11.98 (11.78–12.19)	13.60 (13.40–13.79)	14.00 (13.79–14.22)	2.02 (16.86%)	14.74 (14.56–14.91)	16.51 (16.35–16.67)	16.29 (16.10–16.47)	1.55 (10.51%)	13.94 (13.75–14.12)	15.17 (14.99–15.35)	15.13 (14.93–15.34)	1.19 (8.53%)
85	2.22 (2.12–2.32)	2.53 (2.44–2.63)	2.41 (2.32_2.50)	0.19 (8.56%)	3.45 (3.35.3.55)	3.81 (3.72–3.91)	3.33 (3.24–3.42)	−0.12 (−3.48%)	2.83 (2.73–2.94)	3.15 (3.06–3.25)	2.84 (2.74–2.93)	0.01 (0.35%)
Male												
6	63.12 (62.63–63.61)	66.13 (65.57–66.59)	67.13 (66.58–67.68)	4.01 (6.35%)	66.52 (66.16–66.89)	69.66 (69.34–69.99)	70.24 (69.84–70.64)	3.72 (5.59%)	66.00 (65.62–66.39)	68.54 (68.91–68.17)	68.89 (68.41–69.36)	2.89 (4.38%)
45	26.94 (26.57–27.30)	29.27 (28.94–29.61)	29.91 (29.53–30.29)	2.97 (11.02%)	29.84 (29.56–30.12)	32.29 (32.04–32.53)	32.50 (32.21–32.79)	2.66 (8.91%)	29.42 (29.13–29.71)	31.26 (30.98–31.54)	31.36 (31.03–31.69)	1.94 (6.59%)
65	11.45 (11.18–11.72)	13.36 (13.10–13.61)	13.65 (13.37–13.93)	2.20 (19.21%)	13.73 (13.52–13.95)	15.75 (15.55–15.95)	15.52 (15.30–15.75)	1.79 (13.04%)	13.23 (13.00–13.46)	14.77 (14.55–15.00)	14.65 (14.40–14.91)	1.42 (10.73%)
85	2.21 (2.05.2.37)	2.73 (2.58–2.87)	2.56 (2.43–2.70)	0.35 (15.84%)	3.35 (3.19–3.51)	4.02 (3.88–4.16)	3.39 (3.25–3.53)	0.04 (1.19%)	2.87 (2.71–3.04)	3.41 (3.26–3.56)	3.01 (2.88–3.15)	0.14 (4.88%)
Female												
6	66.71 (66.17–67.26)	68.37 (67.86–68.88)	69.21 (68.61–69.81)	2.50 (3.75%)	71.15 (70.72–71.59)	73.18 (72.80–73.57)	73.18 (72.72–73.64)	2.03 (2.85%)	69.95 (69.49–70.42)	71.12 (70.68–71.56)	71.22 (70.68–71.76)	1.27 (1.82%)
45	29.30 (29.72–28.87)	30.70 (30.30–31.10)	31.43 (30.98–31.87)	2.13 (7.27%)	33.31 (32.96–33.66)	35.10 (34.79–35.42)	34.97 (34.60–35.33)	1.66 (4.98%)	32.21 (32.57–31.84)	33.15 (32.80–33.50)	33.20 (32.79–33.61)	0.99 (3.07%)
65	12.45 (12.16–12.75)	13.82 (13.53–14.10)	14.31 (13.98–14.64)	1.86 (14.94%)	15.61 (15.35–15.88)	17.18 (16.94–17.43)	16.98 (16.69–17.26)	1.37 (8.78%)	14.56 (14.28–14.83)	15.53 (15.26–15.79)	15.55 (15.24–15.87)	0.99 (6.80%)
85	2.22 (2.10–2.35)	2.41 (2.30–5.53)	2.28 (2.17–2.40)	0.06 (2.70%)	3.50 (3.37–3.63)	3.67 (3.55–3.80)	3.26 (3.14–3.39)	−0.24 (−6.86%)	2.81 (2.68–2.94)	2.99 (2.87–3.11)	2.70 (2.57–2.82)	−0.11 (−3.91%)

^a^ Δ Growth rate for the total period, presented in absolute terms with the relative growth rate shown in parentheses.

**FIGURE 2:**
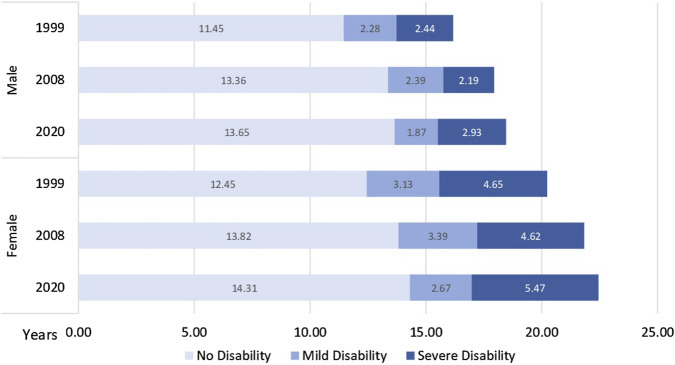
Years with and without disability at 65 years old by sex and period (Spain, 1986–2020).

At age 65, DFLE increased from 11.98 years in 1999 to 14.00 years in 2020 (+2.02 years, +16.9%). SDFLE rose by 1.55 years (+10.5%), and ALE increased by 1.19 years (+8.5%). Increases were also observed at ages 45 and 6, although the magnitude of gains tended to decrease with advancing age. Additionally, at age 65 the composition of years lived with disability shifted, with a relative decrease in mild disability and an increase in severe disability (Figure 2). Between 2008 and 2020, several changes in SDFLE and ALE—particularly at ages 45 and 65—were not statistically significant (overlapping 95% CIs); at age 85, the change in DFLE was also not significant.

When stratified by sex, both men and women experienced increases in DFLE, SDFLE, and ALE between 1999 and 2020. At age 65, DFLE rose by 2.20 years in men (+19.2%) and by 1.86 years in women (+14.9%). SDFLE increased by 1.63 years in men (+13.0%) and by 1.33 years in women (+8.8%). ALE increased by 1.33 years in men (+11.5%) and 1.08 years in women (+6.6%). At age 85, gains were generally smaller and more variable; in some cases, differences were not statistically significant.

Table 4 reports the number of years lived with severe disability and without autonomy. At age 6, years lived with severe disability increased from 4.52 years in 1999 to 4.98 years in 2020 (+0.46 years, +10.2%). At age 65, this value rose from 3.61 to 4.24 years (+0.63 years, +17.5%). Years lived with dependence also increased across all age groups. At age 6, these years rose from 5.38 to 6.63 (+1.25 years, +23.2%). However, for DLY, changes between 1999–2008 and 2008–2020 at ages 6, 45, and 65 were small and not statistically significant. By contrast, most increases in years lived with severe disability and dependence, especially at older ages, were statistically significant.

**TABLE 4:** Years with Disability, Severe Disability and Dependence with 95% Coefficient Intervals at 6, 45, 65 and 85 years old in 1999, 2008 and 2020 (Spain, 1999–2020).

	Years lived with disability				Years lived with severe disability				Years lived with dependence			
	1999	2008	2020	Δ 1999–2020 (abs; %)^a^	1999	2008	2020	Δ 1999–2020 (abs; %)^a^	1999	2008	2020	Δ 1999–2020 (abs; %)^a^
Total												
6	8.44 (8.07–8.81)	8.41 (8.06–8.76)	8.51 (8.10–8.92)	0.07 (0.83%)	4.51 (4.23–4.80)	4.22 (3.97–4.48)	4.97 (4.66–5.28)	0.46 (10.2%)	5.38 (5.07–5.68)	5.82 (5.53–6.11)	6.63 (6.27–6.99)	1.25 (23.24%)
45	7.59 (7.31–7.87)	7.62 (7.36–7.88)	7.68 (7.38–7.97)	0.09 (1.18%)	4.12 (3.90–4.35)	3.90 (3.70–4.10)	4.61 (4.38–4.84)	0.49 (11.89%)	4.89 (4.66–5.13)	5.40 (5.17–5.63)	6.07 (5.80–6.33)	1.18 (24.12%)
65	6.36 (6.16–6.56)	6.43 (6.23–6.62)	6.52 (6.31–6.74)	0.16 (2.52%)	3.61 (3.43–3.78)	3.51 (3.35–3.67)	4.24 (4.05–4.42)	0.63 (17.45%)	4.41 (4.22–4.59)	4.85 (4.67–5.03)	5.39 (5.19–5.60)	0.98 (22.22%)
85	3.45 (3.35–3.55)	3.82 (3.72–3.91)	4.30 (4.21–4.39)	0.85 (24.64%)	2.22 (2.11–2.32)	2.54 (2.45–2.64)	3.38 (3.28–3.47)	1.16 (52.25%)	2.83 (2.73–2.94)	3.20 (3.11–3.30)	3.87 (3.78–3.96)	1.04 (36.75%)
Male												
6	6.76 (6.27–7.25)	6.45 (5.99–6.91)	6.82 (6.26–7.37)	0.06 (0.89%)	3.36 (3.00–3.73)	2.91 (2.59–3.24)	3.70 (3.30–4.11)	0.34 (10.12%)	3.88 (3.49–4.27)	4.04 (3.67–4.41)	5.06 (4.58–5.53)	1.18 (30.41%)
45	5.80 (5.43–6.16)	5.54 (5.20–5.88)	5.87 (5.49–6.25)	0.07 (1.21%)	2.89 (2.61–3.17)	2.53 (2.28–2.77)	3.28 (2.99–3.56)	0.39 (13.49%)	3.31 (3.02–3.60)	3.56 (3.28–3.84)	4.42 (4.08–4.75)	1.11 (33.53%)
65	4.72 (4.45–4.99)	4.58 (4.33–4.84)	4.80 (4.52–5.08)	0.08 (1.70%)	2.44 (2.22–2.66)	2.19 (1.99–2.39)	2.93 (2.70–3.16)	0.49 (20.08%)	2.94 (2.71–3.17)	3.17 (2.94–3.40)	3.80 (3.54–4.05)	0.86 (29.25%)
85	2.81 (2.65–2.97)	2.94 (2.79–3.08)	3.42 (3.29–3.56)	0.61 (21.71%)	1.67 (1.51–1.83)	1.65 (1.51–1.79)	2.60 (2.47–2.73)	0.93 (55.69%)	2.14 (1.98–2.31)	2.25 (2.11–2.40)	2.97 (2.84–3.11)	0.83 (38.79%)
Female												
6	10.08 (9.53–10.63)	10.28 (9.77–10.79)	10.21 (9.61–10.81)	0.13 (1.29%)	5.64 (5.21–6.07)	5.47 (5.08–5.86)	6.24 (5.78–6.70)	0.60 (10.64%)	6.84 (6.38–7.30)	7.53 (7.09–7.97)	8.20 (7.67–8.74)	1.36 (19.88%)
45	9.31 (8.89–9.74)	9.59 (9.19–9.99)	9.48 (9.03–9.93)	0.17 (1.82%)	5.30 (4.95–5.66)	5.19 (4.87–5.50)	5.94 (5.58–6.30)	0.64 (12.08%)	6.41 (6.04–6.78)	7.14 (6.79–7.49)	7.71 (7.30–8.12)	1.30 (20.28%)
65	7.78 (7.48–8.08)	8.02 (7.73–8.30)	8.14 (7.81–8.47)	0.36 (4.63%)	4.62 (4.35–4.88)	4.65 (4.41–4.89)	5.47 (5.19–5.76)	0.85 (18.40%)	5.68 (5.40–5.95)	6.31 (6.04–6.58)	6.90 (6.59–7.21)	1.22 (21.48%)
85	3.79 (3.66–3.91)	4.30 (4.18–4.41)	4.88 (4.76–4.99)	1.09 (28.76%)	2.51 (2.38–2.64)	3.04 (2.91–3.16)	3.90 (3.77–4.02)	1.39 (55.38%)	3.20 (3.07–3.33)	3.72 (3.60–3.84)	4.47 (4.35–4.59)	1.27 (39.69%)

^a^ ΔGrowth rate for the total period, presented in absolute terms with the relative growth rate shown in parentheses.

Across all years, women lived more years with severe disability and dependence than men. At age 6 in 2020, girls lived 6.24 years with severe disability compared to 3.70 years in boys. The same pattern was observed at ages 45, 65, and 85.

## Discussion

This study offers the most comprehensive long-term analysis to date of trends in disability and dependence in Spain, drawing on nationally representative data collected between 1986 and 2020. Over this period, both life expectancy (LE) and disability-free life expectancy (DFLE) increased across all age groups, although not in all subperiods. However, the absolute number of years lived with disability—particularly in its severe and dependent forms—also rose. These findings support the concept of an incomplete compression of morbidity, where gains in healthy life years are not fully offset by reductions in time lived with disability [7, 26].

The observed disability trends are consistent with those reported in earlier periods [15, 16, 27], confirming that the trajectory of DFLE gains, coupled with rising years with disability, has continued into the last decade. This pattern partially aligns with the hypothesis of disability compression proposed by Fries in 1980, which anticipated a decline in disability relative to overall life expectancy [7]. Similar dynamics have been documented in other high-income countries such as Sweden [28, 29], Norway [30], Denmark [31], and the United Kingdoms [32].

One particularly persistent pattern is the gender gap in disability [33], Women consistently exhibit longer life expectancy and longer DFLE than men, but they also live more years with disability. This gap has remained stable in Spain over the study period. The disparity has been attributed to biological factors, historical differences in smoking behaviors, social and economic inequalities, and gender-specific prevalence of disabling and fatal conditions [34, 35]. In the Spanish context, a considerable portion of the sex difference in disability prevalence is explained by higher rates of musculoskeletal disorders among women, notably osteoporosis and osteoarthritis [36]. A relevant hypothesis for future research concerns the long-term effect of smoking uptake among Spanish women since the 1980s [37], and whether this will shift sex patterns in disability over time.

A notable contribution of this study is the observation of an increase in severe disability among older adults, a trend consistent with findings from several high-income countries, particularly in Scandinavia [28, 30, 31]. These results suggest that while some compression of disability may be occurring, it is likely concentrated in milder forms of functional limitation. In contrast, severe disability appears to be expanding, especially in advanced age. This pattern contrasts with Manton’s dynamic equilibrium hypothesis [8], which posits that increased longevity is associated with a shift toward less severe forms of morbidity. Our findings, however, indicate that this equilibrium may not extend to the most disabling conditions. Increases in severe disability among younger age groups appear to be relative rather than absolute, potentially reflecting a postponement in the onset of disabling conditions to more advanced ages. This shift has significant implications for late-life quality of life and long-term care needs.

Alongside the increase in severe disability, this study also documents a rise in dependency among older adults, a pattern not previously observed in Spain. However, similar developments have been reported in countries such as the United Kingdom [38], in Germany [39], and in Japan [40]. These nations have more institutionalized or mixed long-term care systems, where formal services play a stronger role alongside family care. In contrast, Spain represents a Mediterranean, family-based model of care, with a much greater reliance on informal support provided mainly by relatives, particularly women [41]. This combination raises critical concerns about the sustainability of informal support systems in the face of demographic aging, smaller household sizes, and shifting family dynamics.

These findings offer a valuable complement to international indicators such as Healthy Life Years (HLY), which, despite their utility, often rely on simplified measures of limitation and do not distinguish severity or dependency. By using nationally detailed data, this study provides a more nuanced picture of disability dynamics and associated care needs, which are particularly relevant in familialist welfare contexts like Spain.

These changes call for a deeper examination of Spain’s long-standing care model. Traditionally, care for people with disabilities and dependent older adults has been provided within families, predominantly by women [17]. Yet major cultural and structural changes—including greater female labor market participation, aging of caregivers, and evolving family configurations—are challenging this model [42]. Martínez-Pastor [43], distinguishes between familial strategies, where care remains within the household (e.g., through reduced work hours or intergenerational support), and defamilialization, where responsibility shifts toward public services or private care providers. Although these categories were first developed in the context of child care, they are increasingly applicable to elder care.

Recent studies show that most dependent individuals in Spain are still cared for by relatives—especially spouses or children—with a significant gender bias [18]. However, preferences are shifting: nearly 17% of those over 85 now rely on external caregivers, and institutionalization affects about 8% of the disabled population [44, 45]. These trends suggest that defamilialization is gradually expanding, particularly among the oldest age groups, as new forms of care become more socially acceptable and financially viable.

Looking ahead, Spanish society will likely face a widening imbalance between the growing number of individuals requiring care and the shrinking pool of potential informal caregivers. This demographic tension may further accelerate the demand for alternative forms of care provision—whether market-based, community-driven, or publicly supported. Monitoring and anticipating these transitions will be essential for informing long-term care policies that are equitable, resilient, and adapted to demographic realities.

These findings should also be interpreted in the light of international policy frameworks on ageing. The World Health Organization (WHO) defines healthy ageing as the process of developing and maintaining the functional ability that enables wellbeing in older age [5]. According to this perspective, increasing years lived with severe disability and dependence highlight the need to create environments and services that support functional ability throughout the life course. In line with the WHO Decade of Healthy Ageing 2021–2030 [46], our results stress the importance of prevention, rehabilitation, and integrated long-term care policies that allow older adults not only to live longer, but to preserve autonomy and quality of life.

### Strengths and Limitations

This study is based on four large-scale, nationally representative disability surveys conducted over a period of more than three decades. These surveys provide high-quality data, enabling an in-depth analysis of long-term trends in disability and dependence in Spain. A key strength of the study lies in its extended temporal scope, which allows for the comparison of multiple cohorts and the observation of structural changes in disability patterns over time.

However, several limitations should be acknowledged. First, the surveys were conducted in different years and under evolving methodological frameworks. The 1986 survey, in particular, employed a different conceptualization of disability, which required retrospective harmonization to ensure comparability. While efforts were made to standardize definitions and indicators, the questionnaires were not identical across waves, potentially introducing measurement bias. Second, the disability measures used, although based on the International Classification of Functioning (ICF), include survey-specific adaptations. As a result, direct comparability with disability trends in other countries using standardized instruments may be limited. Third, the cross-sectional design of the study, based on four independent time points, restricts causal inference and limits the ability to track individual trajectories of disability over time. Another limitation is that our analysis was restricted to individuals living in private households. While the core samples of all four disability surveys excluded institutionalized populations, supplementary institutional modules were collected in 2008 and 2020. We did not include these in order to ensure comparability across the entire 1986–2020 period, since their design and weighting differ from the main survey samples. As a result, our estimates may underestimate the prevalence of disability and dependence at the oldest ages, where institutionalization is more common, and trends at age 85 may be partly influenced by changes in institutionalization over time.

Despite these limitations, this is the most extensive long-term analysis of disability trends in Spain to date. The study also benefits from the use of official mortality and population data that comply with international quality standards, and from the inclusion of disability data collected by the Spanish National Institute of Statistics using robust sampling strategies. These strengths reinforce the validity and relevance of the findings for understanding the evolution of disability and care needs in aging populations.

### Conclusion

This study provides a comprehensive overview of long-term trends in disability and dependence in Spain over the last four decades, using nationally representative data. The results show that while life expectancy and the number of years lived without disability have increased, there has also been a rise in the number of years lived with disability, particularly in its severe and dependent forms. These patterns are especially evident in older age groups and highlight the growing complexity of aging trajectories.

The persistence of gender disparities in disability, the increase in care needs among the oldest individuals, and the gradual shift in care arrangements from family-based to mixed or formal solutions underscore the importance of rethinking current approaches to long-term care. Spain’s demographic and social transformations call for policies that strengthen the resilience and equity of care systems, ensuring adequate support for people with disabilities throughout the life course.

By documenting changes over an extended time span, this study offers valuable evidence for public health planning and for the development of inclusive and sustainable care policies in aging societies. Future research should further explore how disability trajectories evolve across cohorts, and how social, economic, and institutional factors shape access to care and support.

## Data Availability

All population, mortality, and disability data can be downloaded from the website of the Spanish National Institute of Statistics: https://www.ine.es/.
