# Supplementary material for: Changes in Disability, Severe Disability, and Dependence in Spain (1986–2020)
Source: Int J Public Health. 2025 Sep 29;70:1608931. doi: 10.3389/ijph.2025.1608931 (PMC12515729; doi:10.3389/ijph.2025.1608931)
Supplement: Supplementary file 1 [file Supplementaryfile1.docx]

It must be noted that the conceptualization of disability in the 1986 survey differed from subsequent waves, as it was still based on the 1980 International Classification of Impairments, Disabilities, and Handicaps (ICIDH), whereas the 1999 survey already incorporated draft versions of the International Classification of Functioning, Disability and Health (ICF) available at that time ^1^. In later surveys, the organizers faced a key methodological choice: whether to fully implement the WHODAS 2.0 instrument—thereby aligning completely with the ICF conceptual framework but losing comparability with earlier data—or to replicate previous formats in order to preserve longitudinal consistency. They ultimately adopted a mixed approach. While the ICF conceptual model was used as the basis, the survey instrument remained only partially aligned with WHODAS 2.0. Notably, certain domains such as vision and hearing were retained as disability categories, despite being classified as impairments under the ICF. This compromise was intended to balance conceptual rigor with the need for cross-survey comparability. For further details, see the conceptual work by Abellán ^2^.

Based on this framework, two disability measures were constructed. The first is a **harmonized measure**, restricted to domains available and comparable across all four surveys (vision, hearing, mobility, and self-care) and used in the main trend analyses (see Supplementary Table 1). The second is a **comprehensive measure**, incorporating a wider range of domains, severity levels, and dependency status, but excluding the 1986 survey due to incompatibility (see Supplementary Table 2).

**Supplementary Table 1. Harmonized disability measure in Disability Surveys from 1986, 1999, 2008, and 2020 with survey codes.**

| **Disability dimension** | **EDDM-1986** | **EDDES-1999** | **EDAD-2008** | **EDAD-2020** |
| --- | --- | --- | --- | --- |
| **Vision** | - Total blindness in both eyes (11). - Distinguish images on TV from more than 2 meters, read the newspaper, sew, or see the time on the clock (13). | - Total blindness in both eyes (11). - Perform long-distance visual activities, like identifying objects on the street (12). - Perform short-distance visual activities, like distinguishing TV images from 2 meters away, reading the newspaper, sewing, or telling time on the clock (13). | - Total blindness in both eyes (11). - See the text of a newspaper (21). - See a person's face across the street (31). | - Total blindness in both eyes (11). - See the text of a newspaper (21). - See a person's face across the street (31). |
| **Hearing** | - Total deafness in both ears (21). - Maintain a conversation in a normal tone (23). | - Total deafness in both ears (21). - Hear sirens, alarms, or other loud sounds (22). - Deteriorated ability to maintain a conversation in a normal tone (23). | - Total deafness in both ears (51). - Hear sirens, alarms, or other loud sounds (61). - Maintain a conversation with several people (71). | - Total deafness in both ears (51). - Hear sirens, alarms, or other loud sounds (61). - Maintain a conversation with several people (71). |
| **Mobility** | - Can only move using a wheelchair (61). - Walk, needing help from another person, technical aid, or prosthetics (62). - Climb ten steps in a row without help (71). - Get up or stay seated due to balance problems (113). | - Perform basic body movements, thus bedridden (51). - Perform basic body movements, thus having severe difficulties getting out of bed, standing up, or staying seated (52). - Perform basic body movements, thus having difficulties moving inside the home without help, including wheelchair users (53). - Walk outside the house, including being unable to climb more than ten steps without help (71). - Move outside the house, including using public transport (72). | - Change posture without help or supervision, such as getting up, sitting down, or lying down (181). - Stay in the same position as needed (191). - Walk or move within the home without help or supervision (201). - Walk or move outside the home without help or supervision (e.g., walking on the street without transport, in public or private buildings, etc.) (211). - Move using public transport as a passenger without help or supervision (221). | - Change posture without help or supervision, such as getting up, sitting down, or lying down (191). - Stay in the same position as needed (201). - Walk or move within the home without help or supervision (211). - Walk or move outside the home without help or supervision (e.g., walking on the street without transport, in public or private buildings, etc.) (221). - Move using public transport as a passenger without help or supervision (231). |
| **Self-Care** | - Need help from another person or assistive devices to perform daily living activities such as bathing, dressing, evacuating, eating, or grooming (51). | - Groom and care for appearance (81). - Control needs and use the service (82). - Dress and undress (83). - Eat and drink (84). | - Wash and dry (271). - Perform basic care (e.g., combing hair, cutting nails) (281). - Use the service and control the need to urinate (291). - Use the service and control the need to defecate (301). - Use the service and control menstruation (311). - Dress and undress (321). - Eat and drink (331). | - Wash and dry (271). - Perform basic care (e.g., combing hair, cutting nails) (281). - Use the service (291). - Dress and undress (301). - Eat and drink (311). |

**Supplementary Table 2. Comprehensive disability measure in Disability Surveys from 1999, 2008, and 2020 with survey codes.**

| **Type of Disability** | **EDDES-1999** | **EDAD-2008** | **EDAD-2020** |
| --- | --- | --- | --- |
| **Vision** | - Total blindness in both eyes (11). - Perform long-distance visual activities, like identifying objects on the street (12). - Perform short-distance visual activities, like distinguishing TV images from 2 meters away, reading the newspaper, sewing, or telling time on the clock (13). - Other vision problems (14). | - Total blindness in both eyes (11). - See the text of a newspaper (21). - See a person's face across the street (31). - Other difficulties (e.g., night vision) (41). | - Total blindness in both eyes (11). - See the text of a newspaper (21). - See a person's face across the street (31). - Other difficulties (e.g., night vision) (41). |
| **Hearing** | - Total deafness in both ears (21). - Hear sirens, alarms, or other loud sounds (22). - Deteriorated ability to maintain a conversation in a normal tone (23). | - Total deafness in both ears (51). - Hear sirens, alarms, or other loud sounds (61). - Maintain a conversation with several people (71). | - Total deafness in both ears (51). - Hear sirens, alarms, or other loud sounds (61). - Maintain a conversation with several people (71). |
| **Communication** | - Communicate through speech (31). - Communicate through alternative languages (32). - Communicate through gestures (33). - Communicate through writing (34). | - Speak understandably (81). - Understand what others say (91). - Understand or express in written texts (101). - Understand or express through gestures, symbols, or drawings (111). - Maintain a conversation in spoken, written, or other alternative languages (121). | - Speak understandably (81). - Understand what others say (91). - Understand or express in written texts (101). - Understand or express through gestures, symbols, or drawings (111). - Maintain a conversation in spoken, written, or other alternative languages (121). |
| **Learning** | - Understand, execute, and perform simple orders (43). - Understand, execute, and perform complex orders (44). | - Learn simple tasks, like reading, writing, adding, or subtracting (151). - Perform simple tasks (161). - Perform complex tasks (171). | - Learn simple tasks, like reading, writing, adding, or subtracting (161). - Perform simple tasks (171). - Perform complex tasks (181). |
| **Mobility** | - Change and maintain various body positions (51). - Get up, lie down, remain standing or seated (52). - Move within the home (53). - Carry/transport lightweight objects (61). - Use utensils and tools (62). - Manipulate small objects with hands and fingers (63). - Walk without transport (71). - Move outside the home, including using public transport (72). - Drive own vehicle (73). | - Change posture without help or supervision, such as getting up, sitting down, or lying down (181). - Stay in the same position as needed (191). - Walk or move within the home without help or supervision (201). - Walk or move outside the home without help or supervision (e.g., walking on the street without transport, in public or private buildings, etc.) (211). - Move using public transport as a passenger without help or supervision (221). - Drive motor vehicles (231). - Lift or transport something with hands (241). - Manipulate and move objects (251). - Manipulate small objects with hands and fingers (261). | - Change posture without help or supervision, such as getting up, sitting down, or lying down (191). - Stay in the same position as needed (201). - Walk or move within the home without help or supervision (211). - Walk or move outside the home without help or supervision (e.g., walking on the street without transport, in public or private buildings, etc.) (221). - Move using public transport as a passenger without help or supervision (231). - Drive motor vehicles (241). - Manipulate objects, or lift and move something with hands (251). - Manipulate small objects with hands and fingers (261). |
| **Self-Care** | - Groom and care for appearance (81). - Control needs and use the service (82). - Dress and undress (83). - Eat and drink (84). | - Wash and dry (271). - Perform basic care (e.g., combing hair, cutting nails) (281). - Use the service and control the need to urinate (291). - Use the service and control the need to defecate (301). - Use the service and control menstruation (311). - Dress and undress (321). - Eat and drink (331). | - Wash and dry (271). - Perform basic care (e.g., combing hair, cutting nails) (281). - Use the service (291). - Dress and undress (301). - Eat and drink (311). |
| **Domestic Life** | - Perform and manage shopping (91). - Prepare meals (92). - Clean and care for clothes (93). - Clean and maintain the house (94). | - Perform and manage shopping (361). - Prepare meals (371). - Perform household tasks (e.g., clean or wash clothes) (381). | - Manage shopping (341). - Shop and prepare food (351). - Perform household tasks (e.g., clean or wash clothes) (361). |
| **Interactions** | - Maintain affectionate relationships with family (101). - Start and maintain relationships with friends (102). - Start and maintain relationships with colleagues, bosses, or subordinates (103). | - Show affection (391). - Start or maintain relationships with subordinates, peers, or superiors (411). - Start and maintain relationships with friends or colleagues (421). - Start a family or maintain family relationships (431). | - Show affection (371). - Start or maintain relationships with friends, neighbors, subordinates, peers, or superiors (391). - Start a family or maintain family relationships (401). |

**References**

1. *WHO. International Classification of Impairments, Disabilities, and Handicaps. Geneva: World Health Organization; 1980.* Available at: https://apps.who.int/iris/handle/10665/41003

2. Abellán A, Esparza C, Castejón P, Pérez J. Epidemiología de la discapacidad y la dependencia de la vejez en España. *Gac Sanit*. 2011;25(SUPPL. 2):5-11. doi:10.1016/J.GACETA.2011.07.010
