# Supplementary material for: Changes in Disability, Severe Disability, and Dependence in Spain (1986–2020)
Source: Int J Public Health. 2025 Sep 29;70:1608931. doi: 10.3389/ijph.2025.1608931 (PMC12515729; doi:10.3389/ijph.2025.1608931)
Supplement: Supplementary file 2 [file Supplementaryfile2.docx]

**Supplementary Figure S1. Population pyramids with disability prevalence by sex in 1986, 1999, 2008, and 2020**


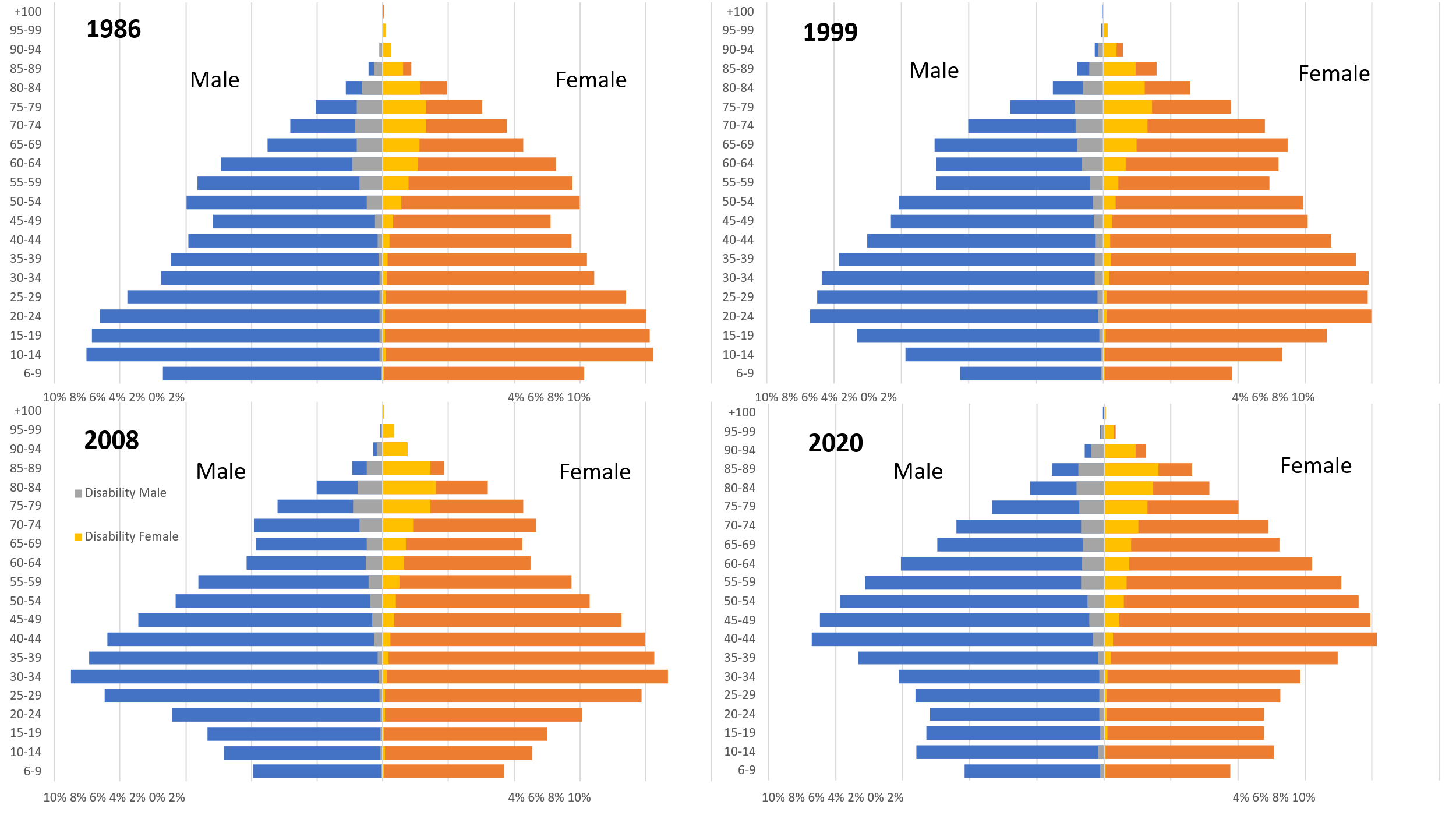


ªHarmonized disability measure to allow comparability with the 1986 survey.
